# An Atypical Presentation of Spondylodiscitis Following a Fall in an Elderly Patient: A Diagnostic Challenge

**DOI:** 10.7759/cureus.113171

**Published:** 2026-07-22

**Authors:** Wala Abbas Obaid, Milikyas A Feyisa, Banan Mohamed Fedail Rajab

**Affiliations:** 1 General Internal Medicine, Naas General Hospital, Naas, IRL; 2 Internal Medicine, Naas General Hospital, Naas, IRL

**Keywords:** atypical back pain, diagnostic delay, discitis-osteomyelitis, geriatric fall, role of mri

## Abstract

Spondylodiscitis is an uncommon but serious infection of the intervertebral disc space. Because its symptoms are often vague, diagnosis can be delayed, particularly in older patients. We describe the case of an 80-year-old woman who presented after an unwitnessed fall with persistent musculoskeletal pain and markedly raised inflammatory markers. Initial investigations, including imaging and microbiological testing, did not identify a clear source of infection. However, a repeat MRI of the spine on day 19 of admission confirmed discitis. Following targeted intravenous antibiotic treatment, she showed a clear clinical and biochemical response. This case highlights how challenging discitis can be to recognize in elderly patients and reinforces the importance of repeat imaging and multidisciplinary collaboration when clinical suspicion remains high.

## Introduction

Spondylodiscitis is an infection of the intervertebral disc space and adjacent vertebral bodies. Globally, the incidence of infectious spondylodiscitis ranges between 2.2 and 7.4 cases per 100,000 people annually. Annual rates span 0.4 to 2.4 per 100,000 in Western Europe, while in Ireland, vulnerability peaks at 9.8 per 100,000 among adults aged 65 and older. The primary etiology involves hematogenous seeding from a distant site, though direct inoculation from trauma or spinal surgery also occurs. *Staphylococcus aureus* remains the predominant causative microorganism isolated, followed by *Streptococcus* species [1,2].

Because early symptoms are subtle and non-specific, the condition is frequently diagnosed late. Diagnosis relies on clinical suspicion, raised inflammatory markers, microbiological evidence, and radiological findings. MRI remains the imaging modality of choice for diagnosing discitis [3]. Multidisciplinary care improves patient outcomes and healthcare efficiency [4]. This case illustrates the diagnostic difficulty of discitis in an elderly patient whose first presentation was attributed to a fall.

## Case presentation

An 80-year-old patient was brought to the emergency department after a fall at home three days before admission. The fall was unwitnessed, and she was found by a family member the following day. She complained of severe pain in her left shoulder and leg. Before this event, she had been living independently and had no history of chronic back pain. Her medical history included hypertension, chronic bilateral leg ulcers, and a previous fall in 2022. On examination, she was afebrile and hemodynamically stable, but her pain was severe enough to limit movement of the left arm and leg. Neurological assessment was limited by pain, although no clear focal neurological deficit was identified.

**Figure 1:**
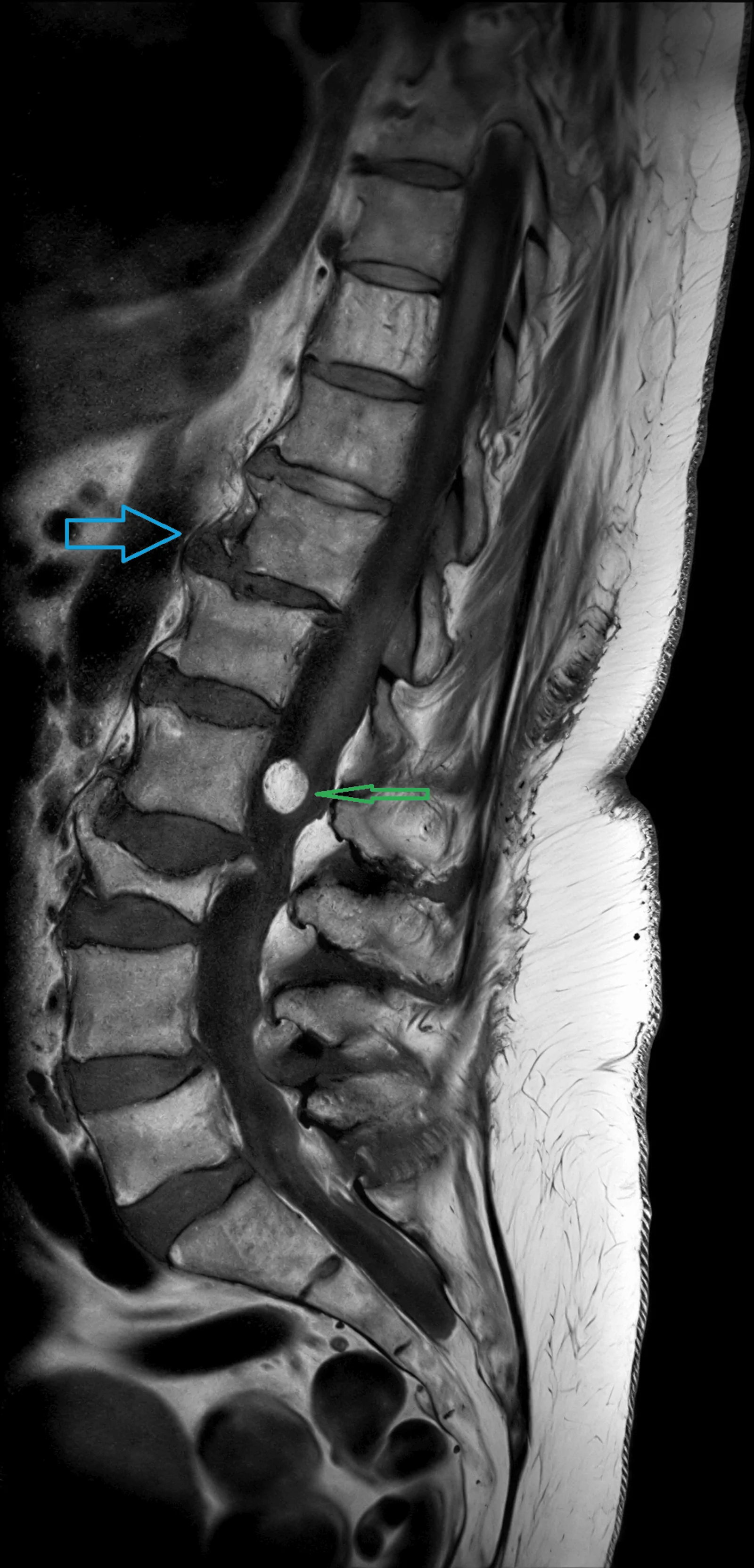
Initial MRI of lumbosacral spine without contrast T12 - L1 disc protrusion indenting the thecal sac indicated with blue arrow, and incidental intradural lipoma of the conus indicated with green arrow

Initial blood tests showed a markedly raised CRP of 240 mg/L (normal < 5 mg/L), which later peaked at 446 mg/L. Creatine kinase was mildly elevated at 508 U/L (normal < 190 U/L). The peripheral blood film supported a picture of systemic inflammation, with toxic granulation and band neutrophils. During admission, liver function tests showed a mild cholestatic pattern, and this was investigated with abdominal ultrasound and magnetic resonance cholangiopancreatography (MRCP), but it didn’t show a hepatobiliary source of infection. Plain radiographs of the shoulder, chest, and knee did not reveal any acute abnormality. CT imaging showed no acute findings, and old lumbar vertebral fractures were noted. An initial spinal MRI without contrast was non-revealing (Figure 1).

**Figure 2:**
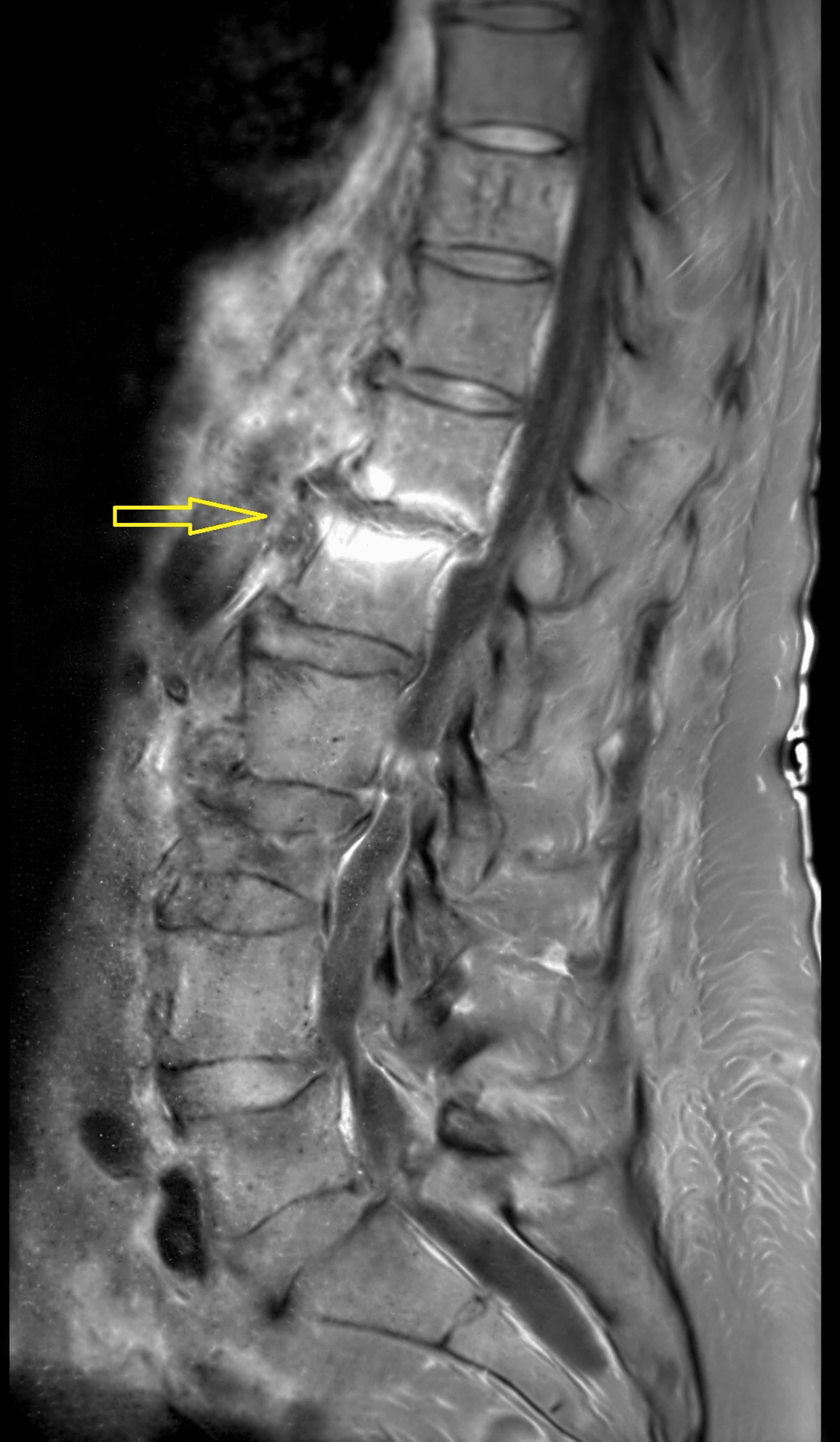
MRI lumbosacral spine with contrast High signal intensity with the adjacent T12-L1 endplate is consistent with interval development of discitis and osteomyelitis (yellow arrow)

She was then managed with intravenous fluids, analgesia, and broad-spectrum antibiotics. Despite this treatment, her pain worsened, mobility declined, and inflammatory markers remained persistently high. A broad search for an infectious source was undertaken, including blood and urine cultures. The echocardiogram showed Cor pulmonale but no evidence of an infective source. Later, antibiotics were temporarily discontinued due to a lack of convincing clinical response. As her symptoms and inflammatory markers persisted, we proceeded with repeat MRI with contrast imaging, which confirmed spondylodiscitis (Figure 2). She was then started on a six-week course of intravenous flucloxacillin and continued with physiotherapy. After targeted treatment, her CRP fell substantially from above 250 mg/L to 62 mg/L, her white cell count normalized, and both her pain and functional status improved significantly, and discharged to home.

## Discussion

The morbidity associated with discitis remains notably high, largely because diagnosis and treatment are frequently delayed. This delay is attributed to the condition’s insidious onset, which can span weeks to months, as well as its non-specific signs and symptoms [5]. Discitis often occurs in immunocompromised individuals-including elderly patients over 65 due to immunosenescence-and may present with misleading chief complaints. In our case, a fall accompanied by back pain created diagnostic confusion, further complicated by other distractors such as gastrointestinal bleeding. These factors collectively contribute to an average diagnostic delay of two to four months for discitis and other spinal infections [5,6].

Magnetic resonance imaging (MRI) remains the gold standard for diagnosing discitis, with a reported sensitivity of 96% and accuracy of 94% [7]. However, MRI performed too early-within 48-60 hours of symptom onset-may fail to detect the infection. The optimal timing for imaging remains a clinical challenge. Based on our literature review, in cases of high clinical suspicion and elevated inflammatory markers after excluding other infectious sources, a repeat MRI with and without gadolinium contrast should be considered 14 days to two to four weeks after an initial negative study. This approach improves diagnostic accuracy and helps distinguish infection from degenerative changes. Two MRI scans performed 14 days apart make spinal infection highly unlikely [7,8].

Early recognition of radiographic features is crucial to avoid diagnostic delays. Plain x-ray findings in early discitis include loss of intervertebral disc space, followed by endplate irregularities, destruction, and annular calcification at the affected level. As the disease progresses, endplate osteopenia appears, along with loss of normal vertebral trabeculation and possible deformity in later stages [8,9]. On MRI, early signs of spondylodiscitis can be subtle and easily mistaken for neoplasm, trauma, other acute inflammatory conditions (such as Modic changes or an acute Schmorl’s node), or degenerative changes, as MR findings may be non-specific or atypical. The earliest response to infection within a vertebral body is the accumulation of extracellular fluid in the bone marrow, seen as blurred margins of inflammatory marrow edema adjacent to an endplate-appearing hypointense on T1WI and hyperintense on T2WI with contrast enhancement [10,11].

## Conclusions

Discitis should remain part of the differential diagnosis in elderly patients who present with persistent back pain, functional decline, and unexplained inflammatory marker elevation, even when there is a history of trauma. Repeat MRI is decisive when the initial imaging is inconclusive. Early recognition, timely targeted antibiotics, and multidisciplinary care are essential for effective management and recovery.
